# Ciliated Muconodular Papillary Tumors of the Lung: Distinct Molecular Features of an Insidious Tumor

**DOI:** 10.3389/fgene.2020.579737

**Published:** 2020-09-29

**Authors:** Xinxin Yang, Yunjing Hou, Jiashi Geng, Jingshu Geng, Hongxue Meng

**Affiliations:** ^1^Department of Pathology, Harbin Medical University Cancer Hospital, Harbin, China; ^2^Department of Radiology, Harbin Medical University Cancer Hospital, Harbin, China

**Keywords:** ciliated muconodular papillary tumors, molecular analysis, histogenesis, immune escape, whole exon gene detection

## Abstract

**Introduction:**

Ciliated muconodular papillary tumors (CMPTs) are rare special peripheral pulmonary nodule composed of different cell proportions, characterized by papillary structures and significant alveolar mucus. Because of their rarity, underrecognized processes, the full range clinical course and histogenesis of CMPTs remains uncertain.

**Methods:**

Molecular features of 5 CMPTs cases (one case with mucinous adenocarcinoma simultaneously) were observed by whole exon gene detection. The histological features of CMPTs and the development trends of three major constituent cells were studied by immunohistochemistry and PCR.

**Results:**

NGS revealed 77 gene mutations in the patient’s tumor tissue and 31 mutations in the border tissue. TMB of CMPT tends to TMB of cancer tissues, and both are higher than normal tissues, CMPT share the same phylogenetic tree with cancer tissues. Moreover, PDL1, B7H3, and B7H4 were overexpressed in high columnar cells and eosinophilic ciliated cells of CMPT, tends to cancer tissues, while LAG3 and siglec15 were not found in CMPT.

**Conclusion:**

The high prevalence of driver gene mutations in CMPTs, similar TMB and phylogenetic tree with cancer tissues indicate their malignant potential. Distinct molecular and immune check point features of each component support the notion that ciliated columnar cells in CMPT are insidious with immune escape.

## Introduction

Ciliated muconodular papillary tumors (CMPTs) are rare peripheral pulmonary nodules characterized by papillary structures and significant alveolar mucus in different proportions. They are composed of a mixture of proliferating ciliated columnar cells, goblet cells, and basal cells surrounded by intra-alveolar mucin pools in the peripheral lung (Kamata et al., 2015; Liu et al., 2016; Taguchi et al., 2017; Chang et al., 2018; Kataoka et al., 2018). Only about 70 cases have been reported worldwide, and the clinicopathological characteristics and histogenesis have not yet been defined in detail. One case of CMPT coexisting with mucinous adenocarcinoma was reported in our cases, it may be a basis for the malignant potential of a CMPT. Through the detection of immune checkpoints, perhaps we can find out the similarities between CMPT and immune escape of malignant tumors.

Recent genetic studies revealed mutations in some driver oncogenes (*BRAF, EGFR, KRAS, AKT1, or ALK*), and they supported the notion that the lesion tends to be a neoplastic lesion with malignant potential (Chuang et al., 2014; Kamata et al., 2015, 2016; Jin et al., 2017; Kim et al., 2017; Taguchi et al., 2017; Udo et al., 2017; Chang et al., 2018; Kataoka et al., 2018). In particular, mutations in *BRAF* (40%) and *EGFR* (30%), as identified by Kamata, support the development of a CMPT as a true tumor process rather than a response or metaplastic disease (Kamata et al., 2016; Table 2). Here, we performed whole exon gene sequencing and immune check point analysis on five CMPT patients to clarify the molecular features and histogenesis of each cell component in CMPT.

## Materials and Methods

### Patients

This study was approved by the institutional review board of Harbin Medical University Cancer Hospital (Harbin, China). Five cases with characteristic features of CMPT were identified between 2016 and 2019. Their clinical and pathologic information were reviewed (Table 1). Tumor tissues and tissues adjacent to cancer were obtained by pathological sampling after surgery. In addition, border tissues beside tumor were enucleated by macrodissection under a stereo microscope.

**TABLE 1:** Characteristics of CMPT patients.

Number	Age	Smoking	Family history	Location	CT finding	Size (mm)	Treatment
1	40–45	–	+	RUL	Ground glass opaque (GGO) nodule	10	Wedge excision
2	60–65	–	–	RLL	Ground glass opaque (GGO) nodule	10	pulmonary lobectomy
3	60–65	+	–	RLL	Ground glass opaque (GGO) nodule	7	Wedge excision
4	60–65	–	–	LLL	Ground glass opaque (GGO) nodule	8	Wedge excision
5	55–60	–	–	RLL	Ground glass opaque (GGO) nodule	10	Wedge excision

*RUL, Right upper lobe; RLL, Right lower lobe; and LLL, Left lower lobe.*

### Immunohistochemistry

Immunohistochemical analysis was performed on formalin-fixed paraffin-embedded (FFPE) sections (4 μm thick) using a fully automated system (Ventana Medical Systems, Tucson, AZ, United States). The slides were stained with antibodies against CK5/6 (clone CK5/6.007, ZSJ-bio, China), thyroid transcription factor-1 (TTF-1) (clone SPT24, Maixin, China), p40 (clone ZR8, Maixin, China), PD-L1 (clone sp22C3, Dako, Japan), B7H3 (Abcam, United States) and B7H4 (Abcam, United States).

### Next-Generation Sequencing

Genomic DNA was sheared into fragments with the size of ∼200 bp. The adapters were added to both ends then were purified with Agencourt AMPure SPRI beads (Beckman Coulter, Inc., Brea, CA, United States). Ligation-mediated PCR was performed to amplify the extracted DNA. For enrichment the PCR products was hybridized to the SureSelect biotinylated RNA library (Agilent Technologies, Santa Clara, CA, United States) according to the manufacturer’s instructions. Paired-end multiplex samples were sequenced with the Illumina HiSeq 2000 System. Sequencing depth was ∼100 × per sample.

### PCR

Total RNA was extracted using an RNeasy Micro kit (Qiagen, Hilden, Germany), then treated according to the manufacturer’s instructions. Complementary DNA (cDNA) was synthesized using a QuantiTect Reverse Transcription Kit (Qiagen). The PCR was performed using cDNA as a template. PCR products were analyzed by 4% agarose gel electrophoresis and stained by ethidium bromide. We used PCR to detect the expression of LAG3 and siglec15 in tissues.

## Results

### Clinical Findings

The patients had a ground glass opacity (GGO) nodule in the lung by chest CT examination, and the size of the nodule in the five patients was generally less than 1 cm. The clinical staging (one case with mucinous adenocarcinoma simultaneously) of the five patients are both T1M0N0. One of them underwent pulmonary lobectomy, and the remaining four cases received wedge excision (Table 1 and Supplementary Figure S1).

### Histologic Findings

**FIGURE 1:**
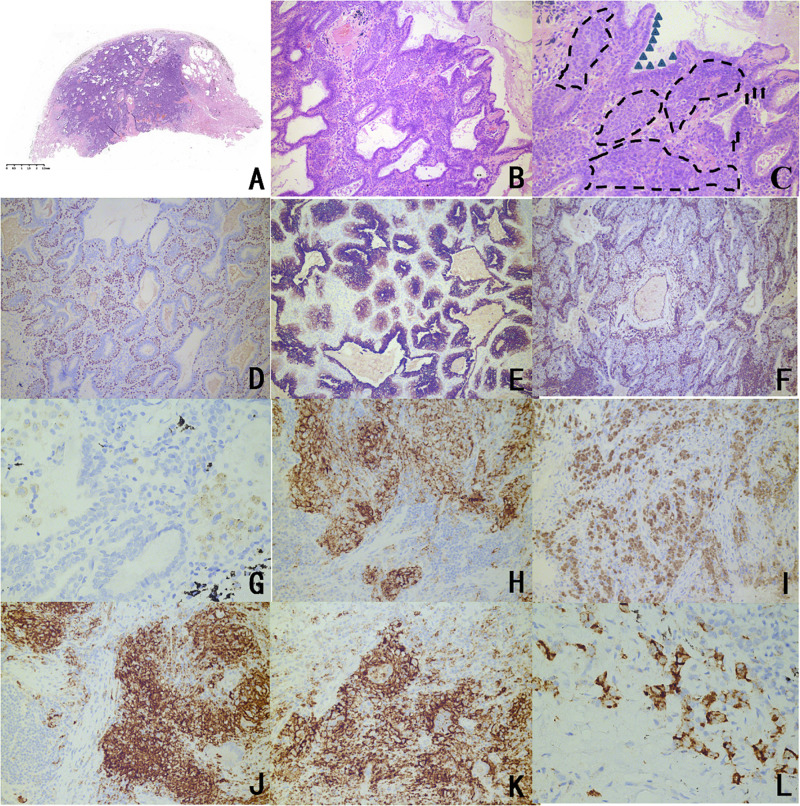
Representative histopathological findings and immunophenotype of CMPTs. **(A)** Microscopic observation revealed a hyperplastic zone with unclear boundaries, and a mucus lake in the alveolar cavity (H&E staining). **(B)** A tubular papillary growth was observed under a medium magnification microscope with chronic inflammatory cell infiltration. **(C)** The microscope was highly magnified, and three main components can be seen in the lesion: basal cells, high columnar cells, and eosinophilic ciliated cells. The triangles are marked as high columnar cells, the arrows are marked mucous cells, and the dashed lines are marked as basal cells. **(D)** TTF-1 of basal cells and columnar cells was stained, and stained stronger than eosinophilic ciliated cells according to immunohistochemistry. **(E)** CK7 staining showed continuous coloring in the basal cells surrounding the adenoid structure and the papillary structure. **(F)** The Ki67 index was less than 5%. Immunohistochemistry for panel **(G)** PDL1 (negative in normal issue); **(H)** PDL1 (positive mainly on high columnar cells and eosinophilic ciliated cells in CMPT); and **(I)** PDL1 (positive in carcinoma). Immunohistochemical analyses of panel **(J)** B7H3, **(K)** B7H4, and **(L)** OFD1 in the CMPTs.

Microscopic observation of the tumor reveals a hyperplastic zone with unclear boundaries, and a mucus lake could be seen in the alveolar cavity. Three main components could be seen under a high powered microscope: basal cells, high columnar cells, and eosinophilic ciliated cells (Figure 1).

### Immunohistochemical Findings

Transcription factor-1 of basal cells and columnar cells was stained, and these cells were stained more than eosinophilic ciliated cells. CK7 staining coloration was continuous in basal cells, and the Ki67 index was less than 5%. Five patients had a positive expression of B7H3, B7H4, PDL1, and OFD1, mainly in high columnar cells and eosinophilic ciliated cells. Among them, adenocarcinoma and CMPT coexisted in the tissue of patient 2, which showed high expression of PDL1 in the CMPT compared to the other four patients, which prompted the activity of immune escape Figure 1 and Table 2).

**TABLE 2:** Summary of immunohistochemical findings from previous reports and the present cases.

Authors	CK5/6	TTF-1	Ki-67	CK7	CK20	MUC5AC	MUC5B	MUC6	MUC2	P53	HNF4α	P63/P40	CDX2	CEA	MUC1	CA125	PDL1	B7H3	B7H4	OFD1
Taguchi et al., 2017	–	0/1	3.7%	1/1	–	1/1	–	1/1	1/1	1/1	–	–	–	1/1	–	–	–	–	–	–
Kataoka et al., 2018	–	4/4	<5%	–	–	3/4	–	2/4	0/4	–	0/4	–	–	–	4/4	4/4	–	–	–	–
Chang et al., 2018	+^*a*^	19/25	–	–	–	–	–	–	–	–	–	+^*a*^	–	–	–	–	–	–	–	–
Kamata et al., 2015)	–	4/4	–	–	–	–	–	–	–	–	–	4/4	–	–	–	–	–	–	–	–
Jin et al., 2017	1/1	1/1	–	1/1	–	–	–	–	–	–	–	1/1	–	–	–	–	–	–	–	–
Udo et al., 2017	–	4/4	<5% 3<10% 1	4/4	0/4	4/4	4/4	–	–	1/4	4/4	4/4	0/4	–	–	–	–	–	–	–
Ishikawa et al., 2016	2/5	3/5	<5% 3<10% 1	5/5	0/5	–	–	–	–	3/3	–	–	–	5/5	–	–	–	–	–	–
Kim et al., 2017	–	1/1	0/1	1/1	–	–	–	–	–	1/1	–	1/1	–	–	–	–	–	–	–	–
Kon et al., 2016	–	5/5	<1% 5	5/5	0/5	0/5	–	0/5	0/5	0/5	–	5/5	–	5/5	5/5	–	–	–	–	–
Chuang et al., 2014	1/1	1/1	<1%	1/1	0/1	–	–	–	–	0/1	–	1/1	–	–	–	–	–	–	–	–
Sato et al., 2010	–	2/2	3%;10%	2/2	0/2	1/2	–	–	–	–	–	–	–	–	–	–	–	–	–	
Our series	5/5	0/5	<5% 5	–	–	–	–	–	–	–	–	5/5	–	–	–	–	5/5	5/5	5/5	5/5

*–, not available. ^*a*^Number of positive/negative not reported.*

### Molecular Findings

**FIGURE 2:**
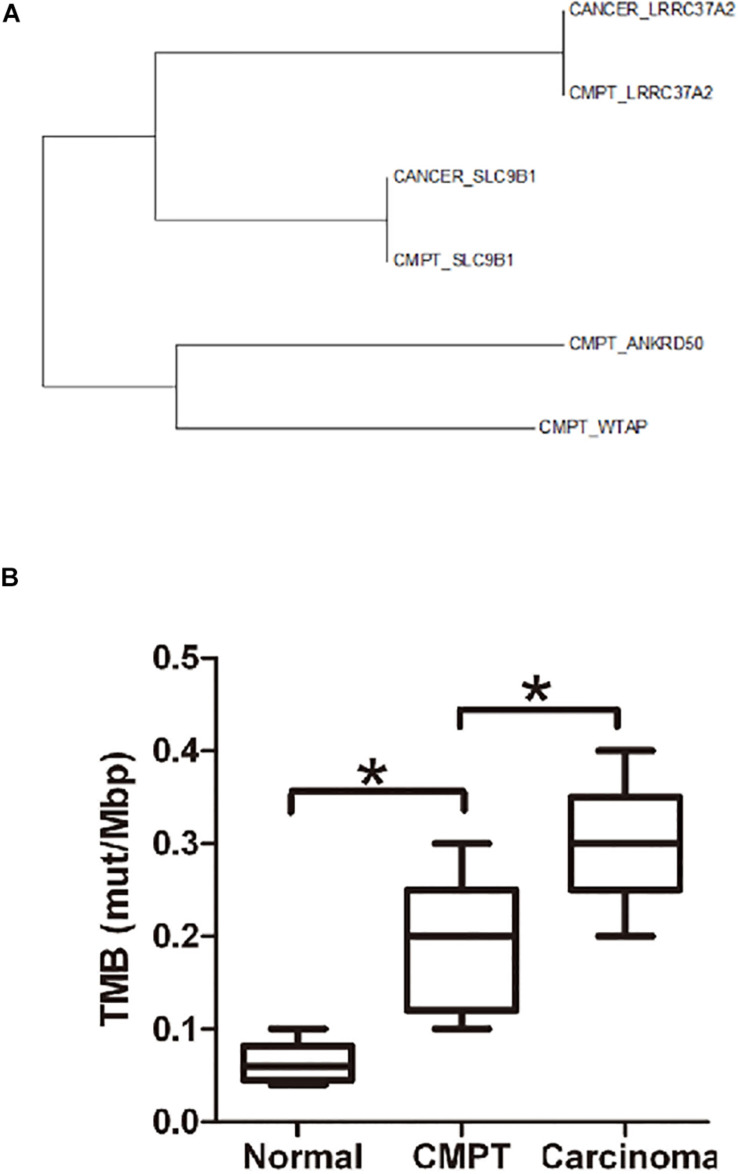
Evolutionary relationships of the taxa and the TMB in different tissues. **(A)** Evolutionary relationships of the taxa. The evolutionary history was inferred using the neighbor-joining method. The optimal tree with the sum of branch length = 2.65875810 is shown. The evolutionary distances were computed using the Poisson correction method and are in the units of the number of amino acid substitutions per site. The analysis involved six amino acid sequences. All positions containing gaps and missing data were eliminated. There were a total of 117 positions in the final dataset. Evolutionary analyses were conducted with MEGA7. **(B)** The TMB (mut/Mbp) in normal tissue, CMPTs, and lung carcinoma. The TMB of CMPT tends to be similar to the TMB of cancer tissues, and both are higher than that in normal tissues. *p < 0.05.

Molecular analysis of NGS revealed 77 gene mutations in the patient’s tumor tissue and 31 mutations in the border tissue (Supplementary Figure S2). We performed a functional enrichment analysis of the tumor variant gene. According to the functional enrichment analysis (Supplementary Figure S3), the first three enriched signal pathways were (1) negative regulation of apoptosis, (2) processing of O-glycogen, and (3) positive regulation of GTPase activity. Both (1) and (3) are associated with excessive proliferation of cells. Furthermore, there were six genes (*EGR1, MUC20, MUC3A, NBPF19, NOL4L*, and *OR4L1*) that were simultaneously mutated in the tumor tissues and junction tissues (Supplementary Figure S4 and Table 3). By analyzing the evolutionary relationship of the taxa, it can be seen that there are three pairs of genes in the same branch of the phylogenetic tree in the CMPT and adenocarcinoma tissues for patient 2. By comparing the TMB in normal tissues and CMPT and adenocarcinoma tissues, it can be seen that the TMB of CMPT is similar to the TMB of cancer tissues, and both are higher than the TMB of normal tissues (Figure 2).

**TABLE 3:** Summary of the detected gene mutations from past reports and the present cases.

Authors	EGFR	BRAF	KRAS	Others
Taguchi et al., 2017	0^*a*^/1^*b*^	0/1	0/1	Alk 1/1
Kataoka et al., 2018	2/4	1/4	1/4	–
Chang et al., 2018	5/21	6/21	4/21	HRAS 1/21
Kamata et al., 2015	–	1/1	–	AKT1 1/1
Jin et al., 2017	–	–	–	ALK 1/1
Udo et al., 2017	–	1/4	1/4	AKT1 1/4
Kim et al., 2017	–	1/1	–	–
Chuang et al., 2014	0/1	–	0/1	–
Kamata et al., 2016	3/10	5/10	–	–
Our series	1/5	–	–	EGR1, MUC20, MUC3A, NBPF19, NOL4L, OR4L1

*–, not available. ^*a*^ Positive number. ^*b*^ Number of people surveyed.*

## Discussion

In this study, we identified a high prevalence of driver gene mutations in the CMPTs; a similar TMB and phylogenetic tree with cancer tissues and an adenocarcinoma coexisted in one case. Distinct molecular and immune check point features of each component provided evidence that these enigmatic lesions are indeed neoplastic processes with immune escape.

Five cases had characteristic features of CMPT, and one of them had mucinous adenocarcinoma simultaneously. Chang et al. (2018) reported one case of CMPT coexisting with adenocarcinoma, and they showed that CMPT may be malignant. The CMPT population has features similar to malignant features, including alveolar structural damage and elastic fiber aggregation, tumor cells proliferating along the alveolar wall, jumping lesions, no capsules, and CEA positivity (Kamata et al., 2015; Kon et al., 2016; Taguchi et al., 2017). Because histology is invasive, a CMPT is easily misdiagnosed as adenocarcinoma with a diagnosis based on frozen pathology. Therefore, we should conduct in-depth research on the cellular components, composition, and developmental trend of CMPT to provide more accurate guidance for clinical work. Chuang et al. (2014) suggested that although CMPT does not meet the criteria for ciliated adenocarcinoma, it has the characteristics of pre-mutation, including goblet (mucus) cell metaplasia and goblet cell TTF-1 staining loss. According to the immunohistochemistry results reported in previous studies, in many cases, CK7/CEA/TTF-1 expressions were positive, and most CK20 expressions were negative. These findings are very similar to those for adenocarcinoma (Sato et al., 2010; Chuang et al., 2014; Ishikawa et al., 2016; Kamata et al., 2016; Kon et al., 2016; Lau et al., 2016; Jin et al., 2017; Kim et al., 2017; Taguchi et al., 2017; Udo et al., 2017; Miyai et al., 2018) and indicate that CMPTs are potential malignant tumors.

Nonetheless, the long-term biological behavior of CMPTs could not be established by the present study, which had a limited follow-up, and larger studies with longer follow-ups are necessary to accurately determine the course of CPMTs (Kamata et al., 2015). Among the five cases, one had a family history of lung cancer, which was her mother (Patient 1), and one case coexisted with lung cancer (Patient 2). This allows us to question whether CMPT really has a malignant potential and whether its subsequent process is lung cancer. Through genetic testing, we identified a high prevalence of driver gene mutations in all CMPTs by whole exon sequencing, and we also found a non-frame shift insertion mutation in exon 20 of *EGFR* in the tumor tissues, which has been considered to be a key driving gene for lung cancer (Supplementary Figure S5). According to the functional enrichment analysis of the tumor variant gene, enriched signal pathways are associated with the excessive proliferation of cells. *MUC20* and *MUC3A* co-mutated at the junction of the tumor and tumor tissues are mucin family genes that are involved in the development of various adenocarcinomas, including lung cancer. Many studies have shown that mucins can be misexpressed in malignant tumors (Zheng et al., 2018). Is this related to the formation of mucus lakes in CMPT? Exploration of more cases is necessary. These results provide a good basis for the tumor properties of CMPT.

Similarly, inconclusive here is whether CMPTs have any potential for malignant transformation with immune escape. We observed the specific influence structure of a CMPT malignant potential and the mechanism of a CMPT malignant potential. We tested the CD28 family of immune escape targets on CMPTs. The expression of PD-L1 (B7H1/CD274), B7H3 (CD276), and B7H4 in tissues was observed in all five patients (Table 2). It is well known that PDL1, B7H3, and B7H4 are highly expressed in tumor tissues to achieve immune escape and promote tumorigenesis (Wiegering et al., 2019). It is notable that PDL1, B7H3, B7H4, and other indicators are mostly expressed in mucus cells of CMPTs. Moreover, we found PDL1 overexpression in CMPTs with adenocarcinoma coexisting compared with other CMPT cases, prompting the presence of immune escape. There is a growing consensus on the importance of PDL1 as a diagnostic biomarker or favorable prognostic factor in CMPTs.

In addition, we also found the high expression of OFD1 in CMPTs by immunohistochemistry, which is an important inhibitor of primary cilia in cancer cells (Wiegering et al., 2019). The elevation of OFD1 indicates a decrease in autophagy and the disappearance of cilia, and studies have shown a close relationship between the disappearance of cilia and tumorigenesis (Tang et al., 2013). The test results for these indicators support CMPTs having a certain malignant potential.

In addition to the CD28 family, we also observed the expression of LAG3 and siglec15 on CMPT tissue, both of which were negative. LAG3 and Siglec15 are novel immunomodulatory targets that inhibit antigen-specific T cell responses, and siglec15 is a major immunosuppressive molecule of PDL1-negative tumors (Nguyen and Ohashi, 2015; Wang et al., 2019). Combined with the CD28 family of immune escape target results, siglec15 negativity coincided with our expectations, and these results support the view that CMPT has malignant potential (Janakiram et al., 2017). Moreover, through biological tree evolution analysis, we found that CMPT and mucinous adenocarcinoma genes share a common evolutionary direction. At the same time, CMPT has the same TMB as adenocarcinoma, and it is higher than that in normal tissue. Among them, high TMB may have a relationship with the gene mutations we detected. This may suggest that mucus cells in CMPT may become cancerous and develop into mucinous adenocarcinoma.

We identified a high prevalence of driver gene mutations in all the CMPTs, a similar TMB and phylogenetic tree as with cancer tissues, and adenocarcinoma coexisted in one case. Distinct molecular and immune check point features of each component provided evidence that these enigmatic lesions are indeed neoplastic processes with immune escape.

## Conclusion

The high prevalence of driver gene mutations in CMPTs, similar TMB and phylogenetic tree with cancer tissues indicate their malignant potential. Distinct molecular and immune check point features of each component support the notion that ciliated columnar cells in CMPT are insidious with immune escape.

## Data Availability Statement

We have uploaded the genetic sequencing data in our manuscript to Dryad Digital Repository.

## Ethics Statement

The studies involving human participants were reviewed and approved by the Institutional Ethics Committee of Harbin Medical University Cancer Hospital (KY2017-27). The patients/participants provided their written informed consent to participate in this study.

## Author Contributions

XY performed the sequence alignment and drafted the manuscript. YH and JiaG carried out the immunoassays. JinG and HM participated in the design of the study and performed the statistical analysis. All authors read and approved the final manuscript.

## Conflict of Interest

The authors declare that the research was conducted in the absence of any commercial or financial relationships that could be construed as a potential conflict of interest.

## Supplementary Material

FIGURE S1 Clinical findings. **(A)** A representative image of gray-whitish tumor was observed in the peripheral lung (arrows) (Patient 1). **(B)** Chest CT shows the right lower lobe ground glass-like nodules (1 × 1 × 1 cm).

FIGURE S2 Mutant genes of tumor and junctional tissues (SNV and Indel).

FIGURE S3 Functional enrichment analysis of mutant genes in tumors.

FIGURE S4 Gene mutation map. Gene mutation map **(A)** EGR1, **(B)** MUC20, MUC3A, **(C)** NOL4L, and **(D)** OR4L1.

FIGURE S5 Mutation site map. Mutation site map of the **(A)** U2AF1.p34F mutation and **(B)** EGFR 20 exon non-frame shift insertion mutation.

FIGURE S6 The mRNA content of PDL1, B7H3 and B7H4 in CMPT and normal tissues. mRNA content **(A)** PDL1, **(B)** B7H3, **(C)** B7H4. **p* < 0.05.

FIGURE S7 Negative control. Negative control **(A)** PDL1, **(B)** B7H3, **(C)** B7H4.

## Abbreviations

CMPTs: ciliated muconodular papillary tumors
FFPE: formalin-fixed paraffin-embedded
GGO: ground glass opaque
TMB: Tumor Mutational Burden
TTF-1: thyroid transcription factor-1.
