# Supplementary figures and images for: Ciliated Muconodular Papillary Tumors of the Lung: Distinct Molecular Features of an Insidious Tumor
Source: Front Genet. 2020 Sep 29;11:579737. doi: 10.3389/fgene.2020.579737 (PMC7550676; doi:10.3389/fgene.2020.579737)

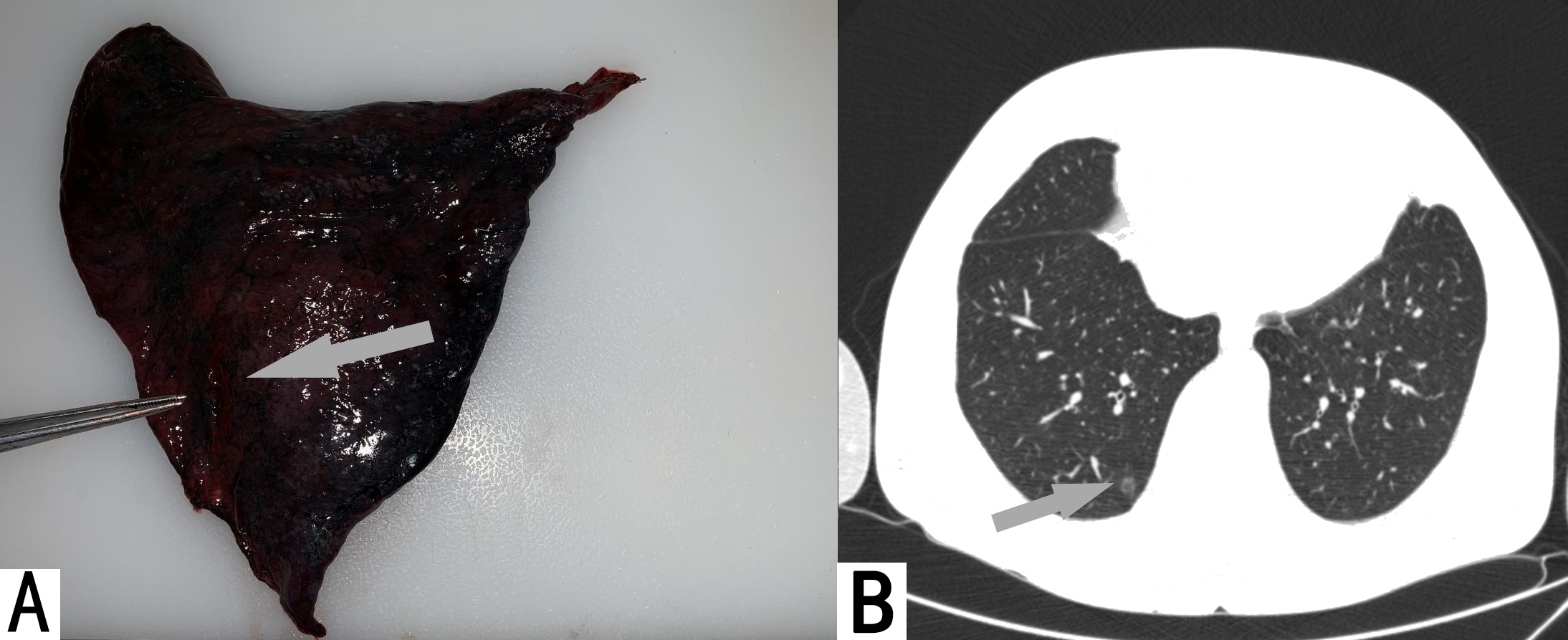

Supplement: FIGURE S1 — Clinical findings. (A) A representative image of gray-whitish tumor was observed in the peripheral lung (arrows) (Patient 1). (B) Chest CT shows the right lower lobe ground glass-like nodules (1 × 1 × 1 cm). [file Image_1.TIF]

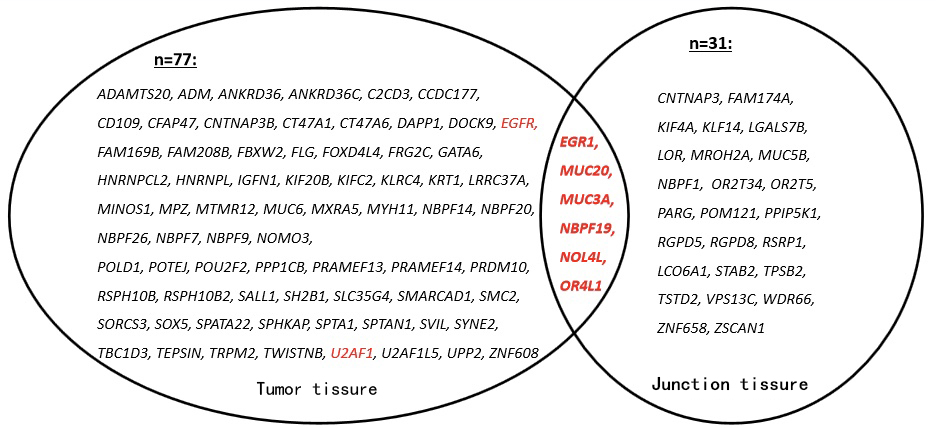

Supplement: FIGURE S2 — Mutant genes of tumor and junctional tissues (SNV and Indel). [file Image_2.TIF]

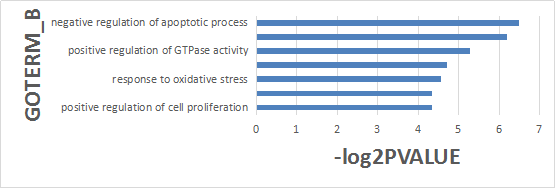

Supplement: FIGURE S3 — Functional enrichment analysis of mutant genes in tumors. [file Image_3.TIF]

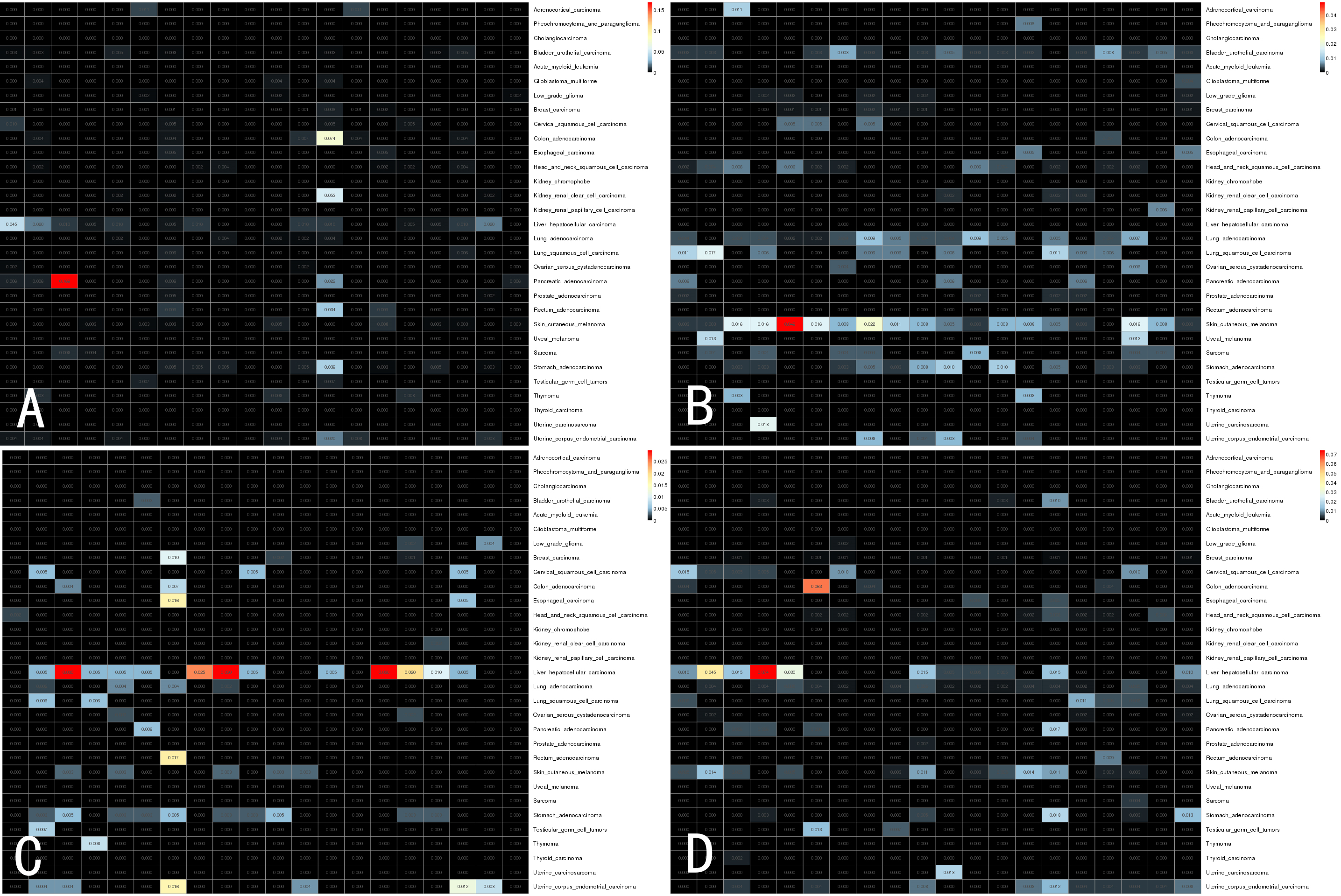

Supplement: FIGURE S4 — Gene mutation map. Gene mutation map (A) EGR1, (B) MUC20, MUC3A, (C) NOL4L, and (D) OR4L1. [file Image_4.TIF]

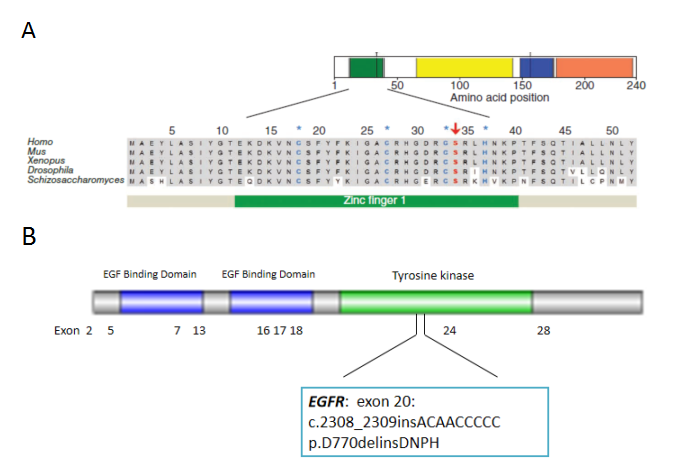

Supplement: FIGURE S5 — Mutation site map. Mutation site map of the (A) U2AF1.p34F mutation and (B) EGFR 20 exon non-frame shift insertion mutation. [file Image_5.TIF]

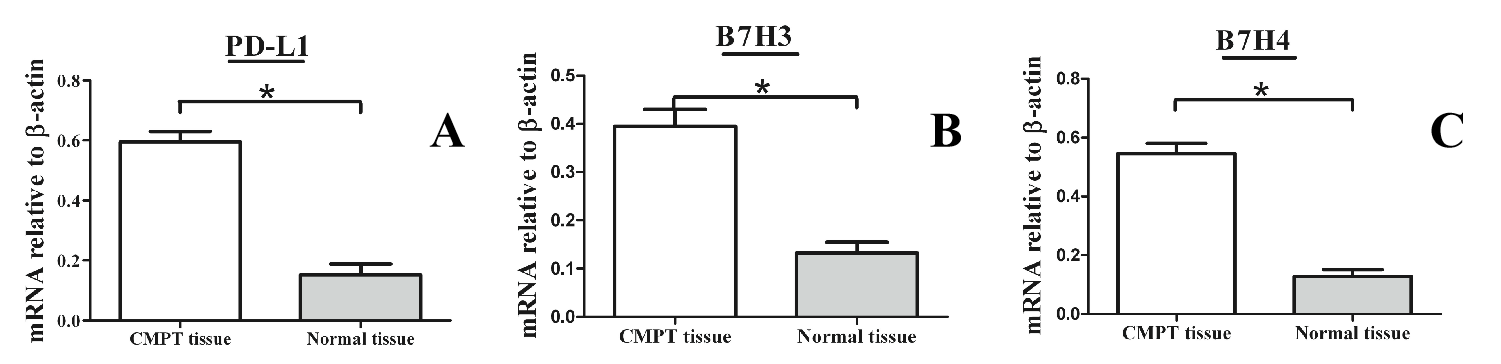

Supplement: FIGURE S6 — The mRNA content of PDL1, B7H3 and B7H4 in CMPT and normal tissues. mRNA content (A) PDL1, (B) B7H3, (C) B7H4. *p < 0.05. [file Image_6.TIF]

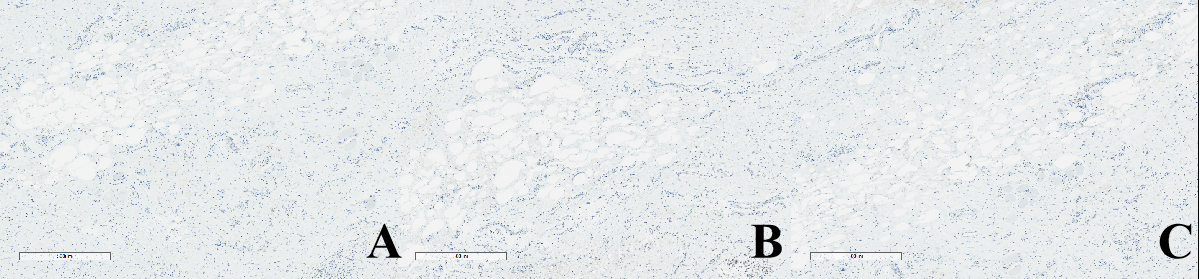

Supplement: FIGURE S7 — Negative control. Negative control (A) PDL1, (B) B7H3, (C) B7H4. [file Image_7.TIF]
